# Genetics reveal the identity and origin of the lionfish invasion in the Mediterranean Sea

**DOI:** 10.1038/s41598-017-07326-1

**Published:** 2017-07-28

**Authors:** Michel Bariche, Periklis Kleitou, Stefanos Kalogirou, Giacomo Bernardi

**Affiliations:** 1 Department of Biology, American University of Beirut, P.O. Box 11-0236, Riad El-Solh, Beirut, 1107 2020 Lebanon; 2 Marine & Environmental Research (MER) Lab, 202 Amathountos Av, Marina Gardens, Block B, Off. 13-14, Limassol, Cyprus; 3 Hellenic Centre for Marine Research, Institute for marine biological Resources and Inland Waters, 190 13 Anavyssos, Greece; 4 Department of Ecology and Evolutionary Biology, University of California Santa Cruz, 100 Shaffer Road, Santa Cruz, CA 95060 USA

## Abstract

Following aquarium releases, invasive lionfishes have colonized large areas of the Caribbean and western Atlantic, resulting in an immense ecological damage. The early stages of that invasion are poorly known. Indeed, a lag of time between the introduction and detection often preclude genetic characterization of that crucial phase. With elevated awareness, the recent invasion of *Pterois miles* was quickly detected in the Mediterranean Sea. We hereby show that the very first individuals establishing populations in the Mediterranean Sea display haplotypes that nest within the large genetic diversity of Red Sea individuals, thus indicating an invasion via the Suez Canal. We also show that only two haplotypes are detected in the Mediterranean Sea, suggesting that few individuals may have been involved in the invasion. Thus, we conclude that the Mediterranean invasion is the result of a movement of individuals from the Red Sea, rather than from other means, and that low genetic diversity does not seem to have a negative effect on the success and spread of lionfish into the Mediterranean Sea.

## Introduction

Invasions of non-indigenous species (NIS) are widely regarded as a major threat to native ecosystems and to global biodiversity^1, 2^. The Atlantic invasion of two lionfish species (*Pterois volitans*, *P. miles*) since the 1990s has been outstanding. Lionfishes have rapidly spread throughout the Western North-Atlantic, the Caribbean Sea and the Gulf of Mexico, reaching very high densities and inflicting important loss of ecological services^3–5^. The native range of the red lionfish, *P. volitans* is the tropical Pacific Ocean, while the congeneric common lionfish, *P. miles* is restricted to the tropical Indian Ocean and the Red Sea^3^, with a narrow overlap in the Indonesian region. The presence of the lionfish in the Atlantic is likely due to human-mediated introduction through the ornamental fish trade^6^ and it is acknowledged that a small founder population originated from aquaria in south Florida^7, 8^. The invasion of the western Atlantic has been disastrous as the invaders directly compete for food resources with native predators and intensively feed on native reef-fishes^4, 9–13^.

*Pterois miles* appeared in the eastern Mediterranean Sea in 2012 and has relatively quickly proliferated and spread, reaching the central Mediterranean Sea^14–20^. A single specimen was recorded 21 years earlier in the eastern Mediterranean Sea^21^ but no other individuals were observed afterwards. Given its conspicuous morphology and significant human presence in the eastern Mediterranean Sea, it is most probable that the recent sightings correspond to a new invasive event^14^. Considering the situation in the western Atlantic, it remains unclear whether the occurrence of the invading population in the Mediterranean Sea is a result of aquarium release, transport by ship ballast water, dispersal event from an Atlantic source or passage through the Suez Canal as Lessepsian immigrant, a major source of NIS introductions in the eastern Mediterranean Sea^22–25^.

The aim of this work is to give insights on the origin and genetic diversity of the early stage of invasion of the non-indigenous lionfish *P. miles* recently established in the Mediterranean Sea. Mitochondrial DNA sequences from the donor (Red Sea) and the recipient regions (Mediterranean Sea)were used to investigate the genetic divergence between regions and possible bottleneck effects. In addition, DNA data from GenBank were used to supplement our data and relate as a control, to its congeneric, *P. volitans*. The current findings are discussed in the light of non-indigenous fish species early invasion stages.

## Results

### DNA sequencing

Fourteen *P. miles* mitochondrial control region sequences were obtained from Lebanon (6), Cyprus (7) and Greece (1). Alignment of control region sequences resulted in 352 base pairs (bp) with no gaps being necessary for perfect alignment. Out of those 352 bp, 10 bp were variable, resulting in two haplotypes. Thirteen individuals were identical and differed from the last individual by 10 nucleotide positions.

### Phylogenetic reconstructions and molecular identity of Mediterranean samples

**Figure 1:**
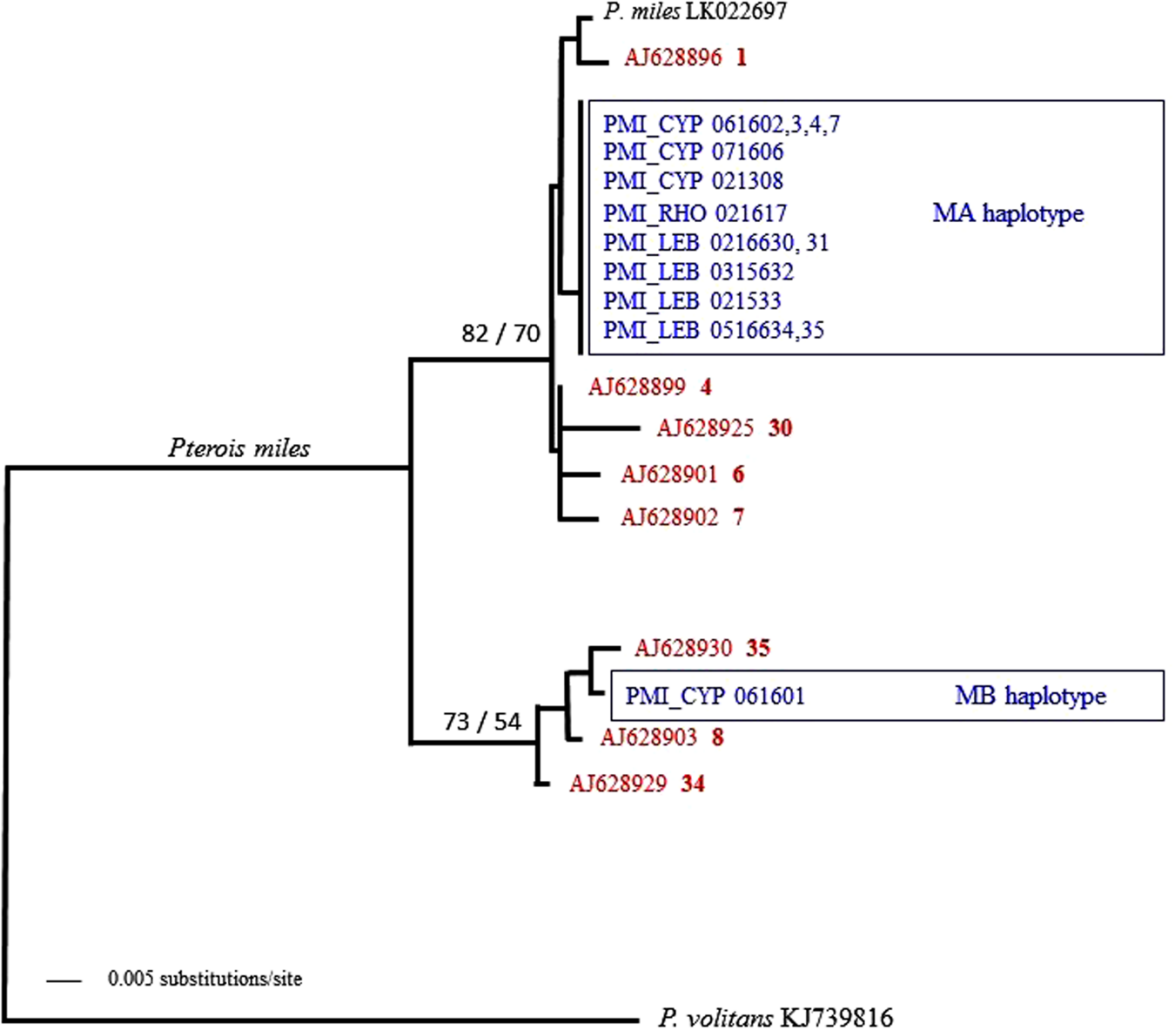
Neighbour-Joining (NJ) tree-based K2P distances of mitochondrial control regions of the common lionfish, *Pterois miles*, from the Red Sea (red, from GenBank) and the Mediterranean Sea (blue, sampled) (identical topology obtained using Maximum Likelihood, ML). Bootstrap support values higher than 50% are shown next to the corresponding nodes where the first value represents the NJ distance and the second value represents the ML value. PMI: *Pterois miles*; CYP, Cyprus; RHO, Rhodes in Greece; LEB, Lebanon. GenBank accession and haplotype numbers are indicated^28^. In black is the control region extracted from a complete mitochondrial genome of *P. miles* from the Gulf of Aqaba and as an out-group from its congeneric *P. volitans*.

To present our results in a broader context, we included previously published lionfish sequences in our analyses. Neighbour-Joining and Maximum Likelihood methods resulted in identical tree topologies (Fig. 1). For reasons of graphical simplification, only eight out of the 13 previously published haplotypes were used (Fig. 1). The inclusion of the remaining five haplotypes, and all sequences available in Genbank (a total of 105 individuals) did not change the tree topology (Supplementary Figure S1).

Sequences from Mediterranean individuals clustered with published sequences assigned to *Pterois miles* with very strong bootstrap support (100% of replicates). This result was due to the presence of only a single nucleotide difference between the GenBank control region (*P. miles*, Red Sea, accession number LK022697) and the sampled haplotype MA, as opposed to 35 changes with the *P. volitans* sequence, a congeneric species to *P. miles* ^26, 27^. Our results were further illustrated by the phylogenetic analysis revealing a close relationship of our Mediterranean individuals to those collected in the Red Sea (Fig. 1).

### Genetic diversity of Mediterranean samples

Mitochondrial control regions from 14 Mediterranean samples resulted in two haplotypes; MA (13 samples) and MB (1 sample) (Table 1, Fig. 1). These haplotypes differed at 10 nucleotide positions, revealing a difference well within the observed range of divergence among *P. miles* individuals collected in the Red Sea^28^. Both haplotypes were very closely related to those previously described as Red Sea haplotypes (Fig. 1; haplotypes 1 and 35^28^).

**Table 1:** Sampling localities, dates sampled, codes used and haplotypes of the lionfish *Pterois miles* in the Mediterranean Sea.

Country/Locality	Date	Code	Haplotype
Lebanon			
Beirut-North	Feb 2016	LEB_0216630	MA
Beirut-North	Feb 2016	LEB_0216631	MA
Batroun	Mar 2015	LEB_0315632	MA
Batroun	Feb 2015	LEB_0215633	MA
Beirut-West	May 2016	LEB_0516634	MA
Beirut-South	May 2016	LEB_0516635	MA
Cyprus			
Protaras, Famagusta	Jun 2016	CYP_061601	MB
Protaras, Famagusta	Jun 2016	CYP_061602	MA
Protaras, Famagusta	Jun 2016	CYP_061603	MA
Protaras, Famagusta	Jun 2016	CYP_061604	MA
Akrotiri bay, Limassol	July 2016	CYP_071606	MA
Zygi, Limassol	Jun 2016	CYP_061607	MA
Amathounta, Limassol	Feb 2013	CYP_021308	MA
Greece			
Rhodes	July 2016	RHO_071617	MA

## Discussion

### Origin of the Mediterranean population

Four main hypotheses may explain the establishment of the lionfish, *Pterois miles*, in the Mediterranean Sea:

1. an accidental introduction by an aquarium release event, similar to the situation observed in the western Atlantic,
2. a larval transport via ballast water through the Suez Canal^25, 29, 30^,
3. a long-distance larval dispersal event from an Atlantic source, or
4. an immigration of individuals through the Red Sea via the Suez Canal (Lessepsian immigrants), as previously described for several other NIS^22, 31^.

While a definite exclusion of the former three hypotheses is not possible, our results strongly favour an arrival through the Suez Canal. All samples analysed nested within samples collected from the Red Sea (where very limited exports for the pet trade occur). The possibility of ship mediated ballast transport was considered as less plausible since only large adults have been reported and not necessarily close to major ports of the Mediterranean Sea^14–20^. Moreover, the fact that lionfish sightings followed the common invasion pattern of Lessepsian immigrants – first established in the Levant, and then gradually spreading into the eastern Mediterranean (stepping stones), is consistent with an invasion of the Mediterranean Sea through the Suez Canal, which also exclude the Atlantic origin possibility. To this end, the most likely explanation for the Mediterranean invasion is therefore an active movement of individuals from the Red Sea into the Mediterranean through the Suez Canal. Our results revealed the presence of two haplotypes that may also indicate the entry of two individuals. Furthermore, the recent sightings in several places in the Mediterranean Sea (Lebanon, Cyprus, Turkey, Greece, Tunisia) in relatively short intervals, also point to a rapid spread in a specific direction rather than multiple isolated introductions. Such a rapid spread is consistent with the presence of the same, most frequent haplotype, in all three sampled localities (Lebanon, Cyprus and Rhodes). That common haplotype was also found in our earliest collection, 2013.

### Haplotype diversity and frequency

While the presence of a population bottleneck in the early phase of invasion, due to founder effects seems intuitive, little evidence in support of this has been found for Lessepsian immigrants^32, 33^. Even for studies based on very few samples and early detection, such as for the parrotfish *Scarus ghobban*, there was no evidence of population bottlenecks as shown by unique haplotypes for each of the five sampled individuals^34^. For the Mediterranean lionfish, only two haplotypes out of 14 samples were found, one being prominent (13 individuals, MA) while the other one (MB) only seen in a single individual. Furthermore, no spatial or temporal partitions were found. The single haplotype MB was found in Cyprus, where the other haplotype MA is also present, and both MA and MB haplotypes were collected in 2016, while the common haplotype MA was collected in all sampling years (2013, 2015 and 2016). However, in a previous study on *P. miles* performed on 88 individuals collected in the Gulf of Aqaba and the northern Red Sea, 13 haplotypes were uncovered thus setting up an expectation of 2.07 haplotypes out of 14 samples^26^. This expectation matches our observed haplotype frequency (2 haplotypes out of 14). Since the standard expectation that a NIS should experience a reduction in genetic diversity has rarely been the case with Lessepsian immigration, the cause could be the multiple entry possibility via the Suez Canal, which acts as a permanent open gateway.

### Significance of repeated introductions

The single individual of *P. miles* reported in 1991 from the Levant^21^ was most possibly a non-successful invasion attempt since no other individual was reported thereafter. The re-appearance of *P. miles* in the Mediterranean Sea, almost two decades later, is almost certainly due to the arrival of new propagules that resulted in a successful establishment. True introduction rates have changed over time in the Mediterranean Sea and the strongest increase detected was for solitary benthic fishes inhabiting hard bottoms and reefs^35^.

A similar pattern of repeated introductions was observed for another highly invasive species in the Mediterranean Sea, the bluespotted cornetfish *Fistularia commersonii*. The first specimen was captured in 1975 but the real establishment and spread started in 2000 and covered the entire Mediterranean Sea^36–38^. The conspicuous morphology of both the lionfish and the bluespotted cornetfish makes their presence in the interim extremely unlikely. Both cases may suggest that the susceptibility of the Mediterranean to biological invasions has been changing as a result of various biotic and abiotic events and processes^39^ and led to the establishment of new propagules from adapted lineages. At a larger scale, the high number of recent records of newly introduced NIS with historically low introduction rates, such as the lionfish, shows a shift in the nature of Lessepsian immigration^35^.

### The future of the lionfish invasion

The lionfish invasion in the western Atlantic has revealed huge detrimental ecological effects on the local ecosystems. While a model predicted that a natural invasion of the Mediterranean Sea by lionfish was unlikely to be successful^24^, and suggested that the Mediterranean lionfish were probably released through means of aquarium trade, our data suggest that this is unfortunately not the case. The recent and rapid expansion of the lionfish in the Mediterranean is therefore alarming and requires the immediate action of all concerned stakeholders in the area.

## Materials and Methods

### Sampling

**Figure 2:**
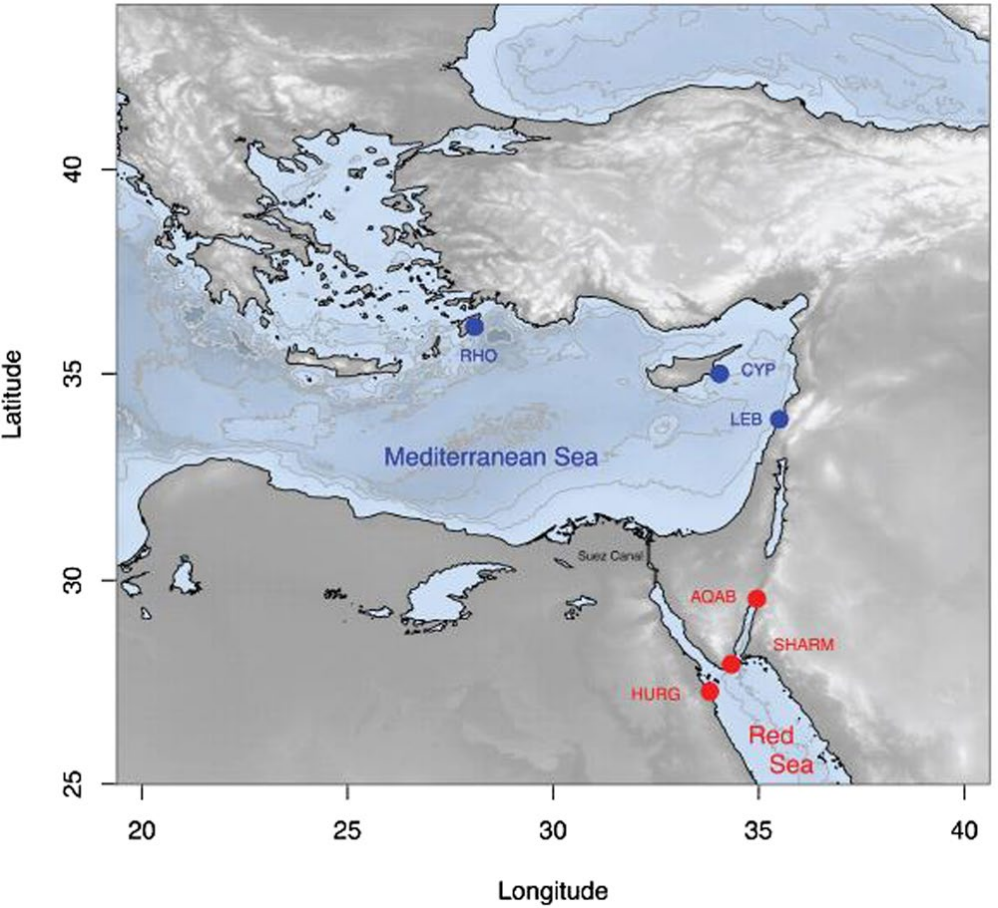
Bathymetric map of the sampling localities in the Mediterranean (blue) and the Red Sea (red): RHO, Rhodes Island in Greece; CYP, Cyprus; LEB, Lebanon; AQAB, Aqaba in Jordan; SHARM, Sharm el Sheik in Egypt; HURG, Hurghada in Egypt. The map was generated using the R package Marmap (0.9.6; cran.r-project.org/)^45^.

Samples were collected for this study by spear or trammel nets from several localities in Lebanon, Cyprus and Greece (Rhodes), between 2013 (one year after its first record in the Mediterranean Sea) and 2016 (Table 1, Fig. 2). They correspond to the very first individuals to be collected from each region. Prior to analysis, all species used in this study were identified following^40^. Fin tissue samples were preserved in 95% ethanol at room temperature. All animal procedures were performed in accordance with UCSC Institutional Animal Care and Use Committee (IACUC - BERNG1601).

### DNA extractions and PCR amplifications

DNA was extracted using standard extraction kits following the manufacturer’s protocol (Blood and Tissue DNA extraction, Qiagen). In order to compare our data with previously published sequences^28^, we amplified and sequenced the mitochondrial control region. Amplification of the mitochondrial control region (also called D-loop) was accomplished with universal primers CR-A and CR-E^41^. Each reaction contained 10 to 100 ng of DNA, 10 mM Tris HCL (pH 8.3), 50 mM KCl, 1.5 mM MgCl2, 2.5 units of Taq DNA Polymerase (Perkin-Elmer, Norwalk, CT), 150 μM of each dNTP, and 0.3 μM of each primer, and was amplified with a cycling profile of 45 s at 94 °C, 45 s at 48 °C, 1 min at 72 °C, for 35 cycles. After purification following the manufacturer’s protocol (ABI, Perkin-Elmer), sequencing was performed with the primers used in the PCR amplification on an ABI 373 automated sequencer (Applied Biosystems, Foster City, CA). To supplement our data, we used GenBank data of sequences for *P. miles* and its congeneric, *P. volitans*, where 13 out of 88 individuals were Red Sea individuals^28^. All sequences from this study were deposited in Genbank under the accession numbers KY713609-KY713622.

### Phylogenetic analyses

Amplification products were sequenced and the computer program Clustal W implemented by Geneious was used to align the mitochondrial control region sequences. Phylogenetic reconstructions were performed based on the Neighbour-Joining method generated in R^42^ with the use of the *ape package* ^43^. Genetic distances were calculated using a Kimura 2 parameter method. We also used the maximum likelihood (ML) method as a second phylogenetic reconstruction approach, as implemented in GARLI^44^. To estimate support for the nodes, 1000 bootstrap replicates were performed and we retained only the values supporting the nodes accounting for more than 50% of the bootstrap replicates.

### Data Availability

14 sequences were deposited at Genbank under the accession numbers KY713609-KY713622.

### Ethical Statement

All animal procedures were carried according to protocols approved by the IACUC at the University of California - Santa Cruz, and all methods were carried out in accordance with relevant guidelines and regulations (IACUC - BERNG1601).

## Electronic supplementary material

Supplementary Figure S1
