# Supplementary material for: Genetics reveal the identity and origin of the lionfish invasion in the Mediterranean Sea
Source: Sci Rep. 2017 Jul 28;7:6782. doi: 10.1038/s41598-017-07326-1 (PMC5533737; doi:10.1038/s41598-017-07326-1)
Supplement: Supplementary file 1 — Supplementary Figure S1 [file 41598_2017_7326_MOESM1_ESM.pdf]

Genetics reveal the identity and origin of the lionfish invasion in the Mediterranean Sea

Michel Bariche*, Periklis Kleitou, Stefanos Kalogirou, and Giacomo Bernardi

Supplementary Figure S1

Neighbour-Joining (NJ) tree-based K2P distances of mitochondrial control regions of the common lionfish, *Pterois miles*, from the Red Sea and the Mediterranean Sea (highlighted in yellow) based on 105 DNA sequences. Sequences from this study are highlighted in yellow. Sequences that are not highlighted correspond to all the best matches found in GenBank against our sequences. The two major clades are labelled according to their assigned species (*Pterois volitans* and *P. miles*).

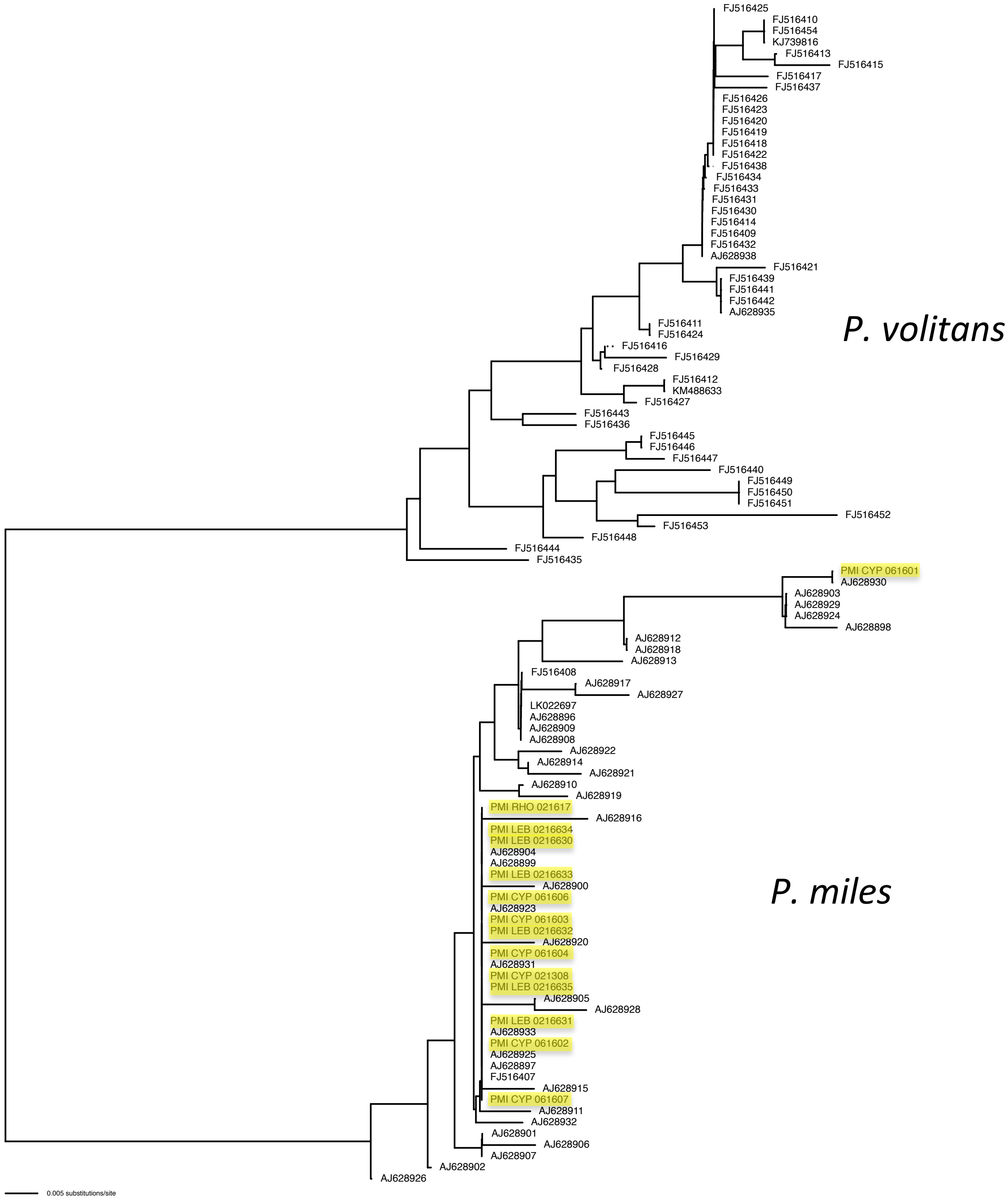
FJ516425
FJ516410
FJ516454
KJ739816
FJ516413
FJ516415
FJ516417
FJ516437
FJ516426
FJ516423
FJ516420
FJ516419
FJ516418
FJ516422
FJ516438
FJ516434
FJ516433
FJ516431
FJ516430
FJ516414
FJ516409
FJ516432
AJ628938
FJ516421
FJ516439
FJ516441
FJ516442
AJ628935
FJ516411
FJ516424
FJ516416
FJ516429
FJ516428
FJ516412
KM488633
FJ516427
FJ516443
FJ516436
FJ516445
FJ516446
FJ516447
FJ516440
FJ516449
FJ516450
FJ516451
FJ516452
FJ516453
FJ516448
FJ516444
FJ516435
P. volitans
PMI CYP 061601
AJ628930
AJ628903
AJ628929
AJ628924
AJ628898
AJ628912
AJ628918
AJ628913
FJ516408
AJ628917
AJ628927
LK022697
AJ628896
AJ628909
AJ628908
AJ628922
AJ628914
AJ628921
AJ628910
AJ628919
PMI RHO 021617
AJ628916
PMI LEB 0216634
PMI LEB 0216630
AJ628904
AJ628899
PMI LEB 0216633
AJ628900
PMI CYP 061606
AJ628923
PMI CYP 061603
PMI LEB 0216632
AJ628920
PMI CYP 061604
AJ628931
PMI CYP 021308
PMI LEB 0216635
AJ628905
AJ628928
PMI LEB 0216631
AJ628933
PMI CYP 061602
AJ628925
AJ628897
FJ516407
AJ628915
PMI CYP 061607
AJ628911
AJ628932
AJ628901
AJ628906
AJ628907
AJ628902
AJ628926
P. miles
0.005 substitutions/site

Supp. Fig. 1
